# Prevalence of parasitism in fecal samples from maned wolves (*Chrysocyon brachyurus*) and domestic dogs in the region of Serra da Canastra National Park, MG

**DOI:** 10.1590/S1984-29612025041

**Published:** 2025-08-07

**Authors:** Daphnne Chelles Marins, Sávio Freire Bruno, Nathalie Costa da Cunha, Luciano Antunes Barros

**Affiliations:** 1 Universidade Federal Fluminense – UFF, Faculdade de Veterinária, Programa de Pós-graduação em Medicina Veterinária (Clínica e Reprodução Animal), Niterói, RJ, Brasil; 2 Universidade Federal Fluminense – UFF, Faculdade de Veterinária, Departamento de Patologia e Clínica Veterinária, Niterói, RJ, Brasil; 3 Universidade Federal Fluminense – UFF, Faculdade de Veterinária, Departamento de Saúde Coletiva Veterinária e Saúde Pública, Niterói, RJ, Brasil

**Keywords:** Canidae, cerrado, conservation, coproparasitological, parasitosis, Canidae, cerrado, conservação, coproparasitológico, parasitose

## Abstract

The Serra da Canastra National Park is a Conservation Unit where domestic dogs live near populations of maned wolves (*Chrysocyon brachyurus*). Recognizing the importance of parasitic diseases in endangered species such as the maned wolf, our research objective was to identify and determine the prevalence of parasites in fresh fecal samples collected in January and July 2021 from both canids that inhabit the region. The dog feces were collected after the administration of a 5% glycerin enema and evacuation, while the maned wolf feces was collected along the park’s roads. For diagnosis, Sheather’s and Hoffman, Pons and Janer (HPJ) parasitological techniques were applied. Among the dog samples, 22.5% (9/40) tested positive for at least one parasite species, mainly 44.4% (4/9) to hookworm eggs, 22.2% (2/9) to *Toxocara* sp. and 11.1% (1/9) to *Cystoisospora* sp. and trematode eggs. Among maned wolves, 75% (30/40) of samples were positive to capillariid eggs with 86.6% (26/30), followed by 16.6% (5/30) to *Toxocara* sp., 10% (3/30) to hookworm eggs and *Dioctophyma renale*, 6.6% (2/30) to *Cystoisospora* sp., trematode eggs and spirurid eggs, 3.3% (1/30) to *Physaloptera* sp. and acanthocephalan eggs.

## Resumo

O Parque Nacional da Serra da Canastra é uma Unidade de Conservação onde cães vivem próximos a populações de lobos-guarás (*Chrysocyon brachyurus*). Reconhecendo a importância das doenças parasitárias em espécies ameaçadas como o lobo-guará, o objetivo desta pesquisa foi identificar e determinar a prevalência de parasitos em amostras fecais frescas coletadas em janeiro e julho de 2021, provenientes de ambas as espécies de canídeos habitantes da região. As fezes dos cães foram coletadas após a administração de enema com glicerina a 5% e evacuação, enquanto as fezes de lobos-guarás foram coletadas ao longo das estradas do parque. Para o diagnóstico, foram aplicadas as técnicas parasitológicas de Sheather e Hoffman, Pons e Janer (HPJ). Das amostras dos cães, 22,5% (9/40) foram positivas para pelo menos uma espécie de parasito, principalmente ancilostomídeos (44,4%, 4/9), *Toxocara* sp. (22,2%, 2/9), *Cystoisospora* sp. e trematódeos (11,1%, 1/9). Entre os lobos-guarás, 75% (30/40) das amostras foram positivas, com predomínio de capilariídeos (86,6%, 26/30), seguidos por *Toxocara* sp. (16,6%, 5/30), ancilostomídeos e *Dioctophyma renale* com 10% (3/30), *Cystoisospora* sp., trematódeos e espirurídeos com 6,6% (2/30), e *Physaloptera* sp. e acantocéfalos com 3,3% (1/30).

## Introduction

In epidemiological studies of wild canids, parasitic diseases are cited as one of the main causes of mortality in maned wolves (*Chrysocyon brachyurus*), which poses problems for the management and recovery programs of populations of this species in their natural habitat. This fact is particularly relevant because this species is under threat of extinction and its population is declining in the biomes where it is endemic, such as the Cerrado and Pantanal (Dinis et al., 1999; Maia & Gouveia, 2002; Paula & Gambarini, 2013)

Environmental degradation and human occupation of the maned wolf’s natural habitat has led to its increasing physical proximity to domestic animals and humans, augmenting the possibility of sharing pathogens, including intestinal parasites. Domestic dogs are known to be affected by several helminths of zoonotic importance, and most of the parasite species found in maned wolf feces have also been found to parasitize humans and/or domestic animals, and are thus considered zoonotic parasites such as *Ancylostoma*, *Trichuris*, *Ascaris*, *Capillaria*, *Toxocara*, *Spirocerca*, *Cystoisospora*, *Giardia*, *Entamoeba*, *Dioctophyma* and *Dirofilaria*, in addition to acanthocephalans and trematodes (Nelson & Couto, 1994; Quinn, 1997; Vicente et al., 1997; Aguirre et al., 2002; Braga et al., 2010; Santos et al., 2012; Paula & Gambarini, 2013; Araújo, 2014; Massara et al., 2015; Dib et al., 2018; Marins et al., 2021).

The Serra da Canastra National Park (PNSC), located in Minas Gerais, is a Conservation Unit and an important remnant of the Brazilian Cerrado, covering approximately 200,000 hectares and has only 8.3% of its area under protection. *C. brachyurus* have individual home ranges reaching up to 134 km^2^. The largest known population of maned wolves is found in the PNSC, with an estimated density of 0.072 individuals/km^2^, and the total Brazilian population is estimated at around 5,642 individuals (Paula & Desbiez, 2014; ICMBIO, 2022, 2024; Lemos et al., 2023).

Despite its wide distribution, the species faces a significant decline due to habitat loss, roadkill, invasive species, illegal hunting, wildlife trafficking, and disease transmission from domestic animals. As a result, the maned wolf is classified as near threatened by the IUCN and vulnerable in Brazil's official red list (Paula et al., 2008; Wilson & Mittermeier, 2009; Aranda et al., 2013; Paula & DeMatteo, 2015; ICMBIO, 2016; Lemos et al., 2023).

The relationship between environment, parasites, and hosts is dynamic and shaped by long-term ecological interactions, with parasites depending on biotic and abiotic factors to complete their life cycles and playing important roles in ecosystem structure and biodiversity. Identifying parasites in fecal samples provides valuable insights into pathogen transmission among hosts, particularly relevant in the context of urban expansion and anthropogenic pressures. However, most studies involving maned wolves rely on fecal analysis with only generic identification of eggs and larvae, due to the rarity of the species and the challenges of obtaining samples (Poulin, 1999; Azpiri et al., 2000; Araújo et al., 2003; Taylor et al., 2017).

Assessing the prevalence of parasites in wild and domestic animals that share habitats is essential for understanding the impact of diseases on wildlife and guiding conservation strategies. Monitoring endoparasitic infections in maned wolves from PNSC and nearby domestic dogs is particularly important for evaluating the health of this endangered species and its role in maintaining ecological balance, supporting both environmental preservation and disease control efforts (Emmons & Feer, 1997; Cleveland et al., 2002).

## Material and Methods

This is an interinstitutional collaborative research study between the Laboratory for Diagnostic Support in Parasitic Diseases at the Faculty of Veterinary Medicine of the Federal Fluminense University, Niterói, RJ, and the Serra da Canastra National Park, São Roque de Minas, MG.

### Study site

The Serra da Canastra National Park (PNSC) covers 200,000 hectares of Cerrado and is located in the southwestern region of Minas Gerais state in Brazil, encompassing the municipalities of São Roque de Minas, Capitólio, Vargem Bonita, São João Batista do Glória, São José do Barreiro, Delfinópolis, and Sacramento. This region is part of the Cerrado biome, recognized as a global biodiversity hotspot, characterized by a tropical rainy climate, with dry winters and rainy summers (Myers et al., 2000; ICMBIO, 2024).

Within the PNSC, altitudes range from 700 m to over 1,400 m, with vegetation classified into forest, savanna, and grassland formations. The specific area where samples were collected for this study is located at approximately 1,300 m in altitude (Ratter et al., 2006).

Maned wolf samples were collected within the PNSC, São Roque de Minas, MG. The collection points within the park were along the main road, starting from Entrance Gate 1 towards the São Francisco River Spring and extending to the upper part of Casca D’Anta Waterfall, covering approximately 27 km.

**Figure 1:**
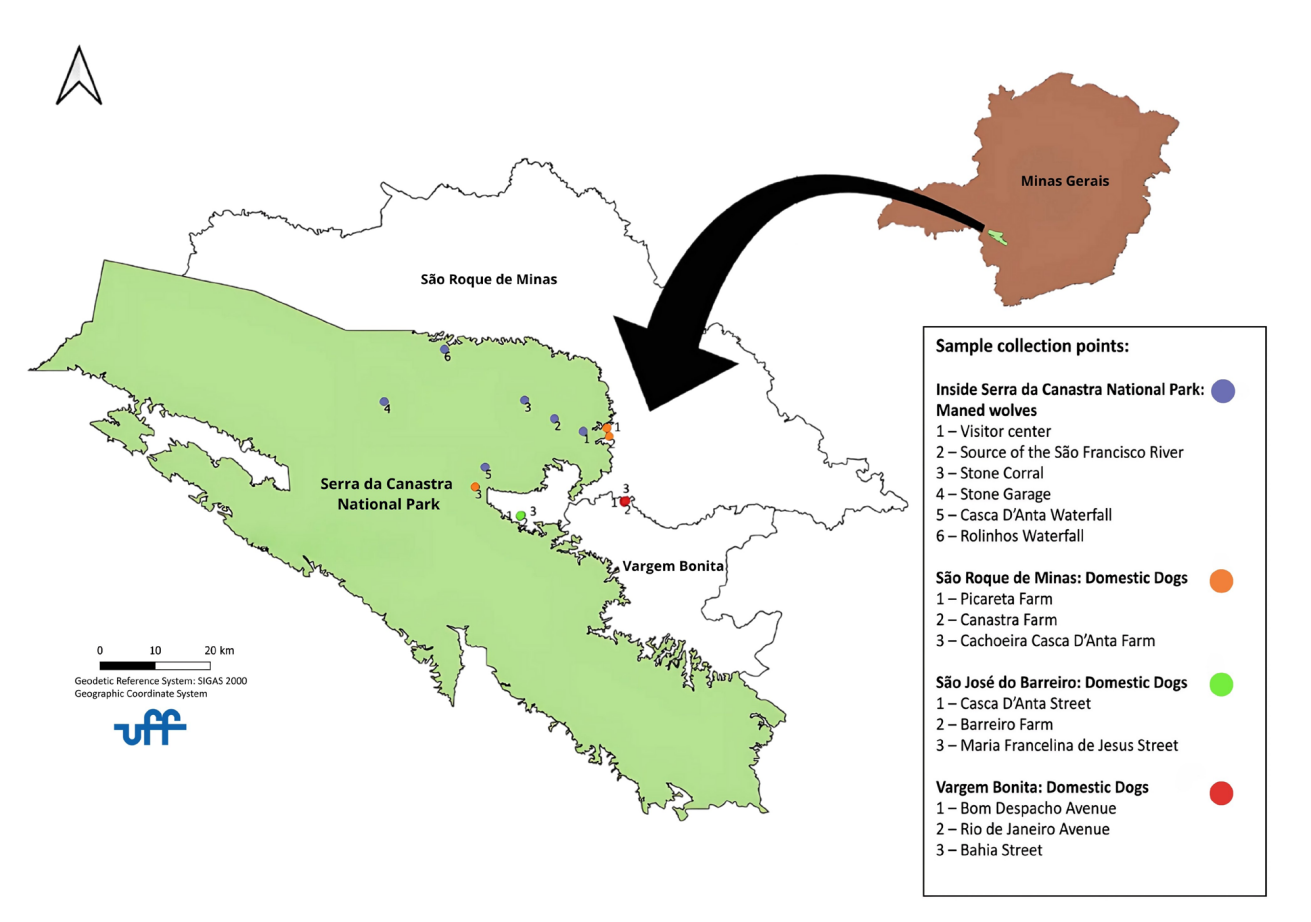
Map showing the fecal sample collection points of maned wolves (*Chrysocyon brachyurus*) and domestic dogs (*Canis familiaris*) in the region of Serra da Canastra National Park, MG. Prepared by Prof. Dr. Flavio Fernando Baptista Moutinho, 2022.

Samples from domestic dogs residing in municipalities adjacent to the PNSC were collected exclusively in rural properties, where maned wolves and domestic dogs share the same habitat. These locations included São Roque de Minas, São José do Barreiro, and Vargem Bonita. The geographic coordinates are available in Table 1, and the map showing the fecal sample collection points is presented in Figure 1.

**Table 1:** Location of fecal sample collections from maned wolves (*Chrysocyon brachyurus*) and domestic dogs (*Canis familiaris*) in the region of Serra da Canastra National Park, MG.

**Species**	**Location**	**Collection point**	**Geographic coordinates**	
*Chrysocyon brachyurus*	Serra da Canastra National Park	Visitor center	20º15’21”S	46º25’00”W
*Chrysocyon brachyurus*	Serra da Canastra National Park	Source of the São Francisco River	20º14’33”S	46º26’49”W
*Chrysocyon brachyurus*	Serra da Canastra National Park	Stone Corral	20º13’23”S	46º28’42”W
*Chrysocyon brachyurus*	Serra da Canastra National Park	Stone Garage	20º13’29”S	46º37’33”W
*Chrysocyon brachyurus*	Serra da Canastra National Park	Casca D’Anta Waterfall	20º17’36”S	46º31’11”W
*Chrysocyon brachyurus*	Serra da Canastra National Park	Rolinhos Waterfall	20º10’11”S	46º33’45”W
*Canis familiaris*	São Roque de Minas	Camping Picareta	20º15’08”S	46º23’31”W
*Canis familiaris*	São Roque de Minas	Canastra Farm	20º15’40”S	46º23’22”W
*Canis familiaris*	São Roque de Minas	Cachoeira Casca D’Anta Farm	20º18’51”S	46º31’48”W
*Canis familiaris*	São José do Barreiro	Casca D’Anta Street	20º20’42”S	46º28’59”W
*Canis familiaris*	São José do Barreiro	Barreiro Farm	20º20’38”S	46º28’53”W
*Canis familiaris*	São José do Barreiro	Maria Francelina de Jesus Street	20º20’39”S	46º28’57”W
*Canis familiaris*	Vargem Bonita	Bom Despacho Avenue	20º19’44”S	46º22’26”W
*Canis familiaris*	Vargem Bonita	Rio de Janeiro Avenue	20º19’50”S	46º22’25”W
*Canis familiaris*	Vargem Bonita	Bahia Street	20º19’43”S	46º22’19”W

### Animals

#### Domestic dogs

Forty different domestic dogs, comprising 60% (24/40) males and 40% (16/40) females, over four months of age, with or without a defined breed, residing on rural properties surrounding the Serra da Canastra National Park, MG, were included in the study. The animals were distributed among the municipalities as follows: Vargem Bonita with 35% (14/40) of the sampled animals, São Roque de Minas with 32.5% (13/40), and the district of São José do Barreiro with 32.5% (13/40).

Nineteen samples were collected in January 2021 (rainy season), and the others 21 samples in July 2021 (dry season), representing two well-defined climatic periods of the Brazilian Cerrado. According to climatological data from the region, average rainfall in January ranges from 250 to 350 mm, while in July it drops significantly to between 5 and 20 mm, reflecting the marked seasonality of the Cerrado biome.

The animals were included in the study after their respective owners signed the Free and Informed Consent Form for the collection of biological material (feces) and the physical clinical examination of the animals. Each animal was registered on an individual clinical record containing clinical data, information about the environment and management, and a physical examination.

The medical history and anamnesis of all dogs were evaluated, followed by a complete physical examination using clinical assessment methods such as inspection, palpation, percussion, olfaction, and auscultation of all organ systems. The findings were recorded in individual follow-up records. The Body Condition Score (BCS), as proposed by Laflamme (1997), was used to assess the dogs' body condition.

All procedures were performed by three veterinarians. The dogs were physically restrained using muzzles to prevent accidents and were placed on a portable examination table to ensure ergonomic comfort for both the team and the animals.

#### Maned wolf

The maned wolves in this study are wild animals of the Brazilian fauna inhabiting the region of Serra da Canastra National Park, MG.

### Fecal samples from domestic dogs

Forty samples were collected from domestic dogs living on properties located in the surroundings of PNSC, encompassing the municipalities of Vargem Bonita, São Roque de Minas, and São José do Barreiro (São Roque de Minas district). All the domestic dogs had owners and could roam around in areas commonly used by maned wolves.

Fecal sample collection was performed by stimulating defecation through the administration of a glycerin rectal enema. The enemas were preassembled using 10 cm segments of plastic tubing (fluid therapy set) attached to 20 mL plastic syringes, each containing 5 mL of 5% pharmaceutical-grade glycerin. This procedure facilitated sample collection and minimized the risk of external contamination, since the samples were always collected only from the most superficial part of the fecal matter immediately after defecation.

### Fecal samples from maned wolves

Forty fecal samples from maned wolves were collected in PNSC. Fresh fecal samples from maned wolves were collected at different points along the main road of the PNSC, starting at one of the entrance gates to the Park (Gate 1) and heading towards the source of the São Francisco River at the upper part of the Casca D’Anta Waterfall. All maned wolf fecal samples collected in the field were georeferenced using a Garmin Etrex 20x GPS, and the collection points are presented in Table 1.

The parameters used to identify fecal samples of wolves were chosen defecation site, presence of footprints in the surrounding area, and presence of traces of their food diet. The fecal samples were collected at dawn, when they were still fresh in the early morning hours at 5:00 to 7:00 a.m.,and only the most superficial part of the feces was removed in order to reduce the risk of contamination.

Of the 40 samples collected, it is not possible to confirm that they all belong to different maned wolves, as the animals were not captured for fecal sample collection, unlike the domestic dogs in this study. Therefore, the possibility of collecting samples from the same individual on different days and seasons of the year was considered. However, no more than one sample was collected per day at the same location, in order to avoid collecting multiple samples from the same individual.

### Parasitological techniques

The fecal samples from both canid species were stored in plastic pots, properly identified, containing liquid preservative (ethyl alcohol 70º GL), placed in a thermal box in room temperature and taken to the Laboratory for Diagnostic Assistance of Parasitic Diseases (LADDP) at the Fluminense Federal University (UFF), where they were processed by the Sheather (centrifugal flotation in saturated sucrose solution) and Hoffman, Pons and Janer (HPJ) (simple sedimentation) techniques.

Three slides of each sample were prepared and examined under an Olympus CX 22 LED optical microscope. The parasitic forms were diagnosed as described by Sloss et al. (1999), and the keys proposed by Yamaguti (1961) and Anderson et al. (1983) were also used. The coefficient of prevalence of fecal parasites was calculated for both host species.

### Data analysis

The dogs’ clinical data were transferred to Excel spreadsheets, and the copro-parasitological test results of the two canid species were subjected to a correlation analysis. A statistical association analysis of the variables was performed, considering solely dogs, solely maned wolves, and the two canid species. Fisher’s Chi-square or Exact test were used when necessary for two independent samples, considering 95% significance. The analyses were performed using the BioStat version 5.3 statistical package (Ayres et al., 2007). The term prevalence was used according to Bush et al. (1997).

## Results

### Epidemiological analysis

#### In domestic dogs

Among the 40 dogs examined, 22.5% (9/40) were infected with at least one parasitic species. Parasitized dogs showed a prevalence rate of 35% (14/40) in the municipality of Vargem Bonita of the PNSC while this rate was 32.5% (13/40) in the both the municipality of São José do Barreiro and of São Roque de Minas. The prevalence rate among males was 60% (24/40) and among females 40% (16/40).

No statistically significant difference was found in the analysis of prevalence rates between male and female dogs (p=0.07171) or in samples from the collection sites, with São Roque de Minas and Vargem Bonita showing p=0.2087, São Roque de Minas and São José do Barreiro p=0.3864, and Vargem Bonita and São José do Barreiro p=1.0000.

#### Dog’s clinical condition

The clinical examinations indicated that approximately half of the dogs had a good body score according to the methodology used for measuring the Body Condition Score proposed by Laflamme (1997), i.e., 55% (22/40), and 80% (32/40) have received anti-parasite medications in the period of 12 months prior to the collection of fecal samples. The medications used in the dogs were based on Pyrantel Pamoate combined with Praziquantel and Febantel, Fenbendazole, Mebendazole, Ivermectin, and Milbemycin oxime combined with Praziquantel.

#### Dog’s Deworming

Regarding the deworming history of the dogs and ectoparasite control, it was found that most dogs were dewormed up to one year before sample collection, with a prevalence of 55% (22/40) of dewormed animals, followed by 25% (10/40) of dogs that were dewormed more than one year prior.

Of the eight dogs that had never been dewormed, four tested positive for parasites. Among the ten dogs dewormed more than a year before this study, three were parasitized, whereas of the 22 dogs dewormed within a year before the study, only two were parasitized.

The prevalence of dogs that had never been dewormed and tested positive for at least one parasite species was 50% (4/8), whereas among dewormed dogs, the prevalence was 15.62% (5/32). Statistical analysis indicated a significant difference (p=0.0294) between animals dewormed within a year before the study and those never dewormed. However, no significant differences were found between dogs dewormed within a year before the study and those dewormed more than a year prior (p=0.2926), nor between dogs dewormed more than a year before the study and those never dewormed (p=0.6305).

### Parasitological analysis

#### Fecal samples of domestic dogs

Of the total number of infected dogs, 44.4% (4/9) were positive for hookworm eggs 22.2% (2/9) for *Toxocara* sp., 11.1% (1/9) for trematode eggs and 11.1% (1/9) for *Cystoisospora* sp. (Table 2). Among the samples positive for parasites, 88.9% (8/9) corresponded to infection by a single parasite and 11.1% (1/9) to infections by multiple parasites, showing a statistically significant difference (p=0.0034).

**Table 2:** Prevalence of sampled dogs submitted to coproparasitological analysis in areas surrounding Serra da Canastra National Park, MG.

**Location**	**Male dog**	**Female dog**	**Prevalence of positives**	**Hookworm eggs**	**Trematode eggs**	***Cystoisospora* sp.**	***Toxocara* sp.**
Vargem Bonita	42.8% (6/14)	57.2% (8/14)	5% (2/40)	0	2.5% (1/40)	0	0
São José do Barreiro	76.9% (10/13)	23.1% (3/13)	5% (2/40)	2.5% (1/40)	0	0	0
São Roque de Minas	61.5% (8/13)	38.5% (5/13)	12.5% (5/40)	7.5% (3/40)	0	2.5% (1/40)	5% (2/40)
TOTAL	60% (24/40)	40% (16/40)	22.5% (9/40)	10% (4/40)	2.5% (1/40)	2.5% (1/40)	5% (2/40)

The prevalence of samples positive for parasites was 25% (2/8) in the dry season and 21.87% (7/32) in the rainy season. A statistical analysis indicated no significant difference (p=1.0000) between the two seasons of the year for parasitism among the dogs.

#### Fecal samples of maned wolves

The prevalence of fecal samples from maned wolves positive for at least one parasite species was 75% (30/40). The most prevalent parasite was capillariid eggs with 86.6% (26/30), followed by *Toxocara* sp., with 16.6% (5/30), hookworm eggs and *Dioctophyma renale* with 10% (3/30), *Cystoisospora* sp., trematode eggs and spirurid eggs with 6.6% (2/30), and acanthocephalan eggs and *Physaloptera* sp. with 3.3% (1/30) (Table 3).

**Table 3:** Prevalence of maned wolf (*Chrysocyon brachyurus*) feces positive for parasitic infections during the rainy and dry seasons in the year 2021, in Serra da Canastra National Park, MG.

Parasites	Prevalence rate	Rainy season	Dry season
Capillariid eggs	86.6% ^a^ (26/30)	26.6% (8/30)	60.0% (18/30)
*Toxocara* sp.	16.6% ^b^ (5/30)	6.6% (2/30)	10.0% (3/30)
Hookworm eggs	10.0% ^b^ (3/30)	-	10.0% (3/30)
*Dioctophyma renale*	10.0% ^b^ (3/30)	-	10.0% (3/30)
*Cystoisospora* sp.	6.6% ^b^ (2/30)	-	6.6% (2/30)
Spirurid eggs	6.6% ^b^ (2/30)	-	6.6% (2/30)
Trematode eggs	6.6% ^b^ (2/30)	-	6.6% (2/30)
Acanthocephalan eggs	3.3% ^b^ (1/30)	-	3.3% (1/30)
*Physaloptera* sp.	3.3% ^b^ (1/30)	-	3.3% (1/30)

Different letters on the lines indicate a statistical difference (p<0.05).

Of the 40 samples examined, 19 were collected during January 2021 (rainy season), with a prevalence of 27.5% (11/40) of positive samples, and 21 samples were collected during July 2021 (dry season), with a prevalence of 47.5% (19/40). The two seasons showed a statistically significant difference (p=0.017481) in parasitism.

**Figure 2:**
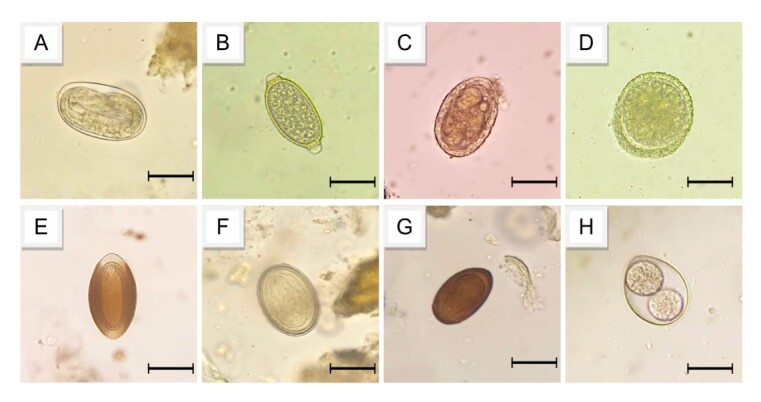
Parasitic structures found in fecal matter from maned wolves (*Chrysocyon brachyurus*) in the region of Serra da Canastra National Park, MG. (A) Hookworm egg, bar = 20 μm; (B) Capillariid egg, bar = 20 μm; (C) *Dioctophyma renale*, bar = 20 μm; (D) *Toxocara* sp., bar = 20 μm; (E) Acanthocephalan egg, bar = 20 μm; (F) Spirurid egg, bar = 20 μm; (G) Trematode egg, bar = 20 μm. H) *Cystoisospora* sp., bar = 50 μm.

**Figure 3:**
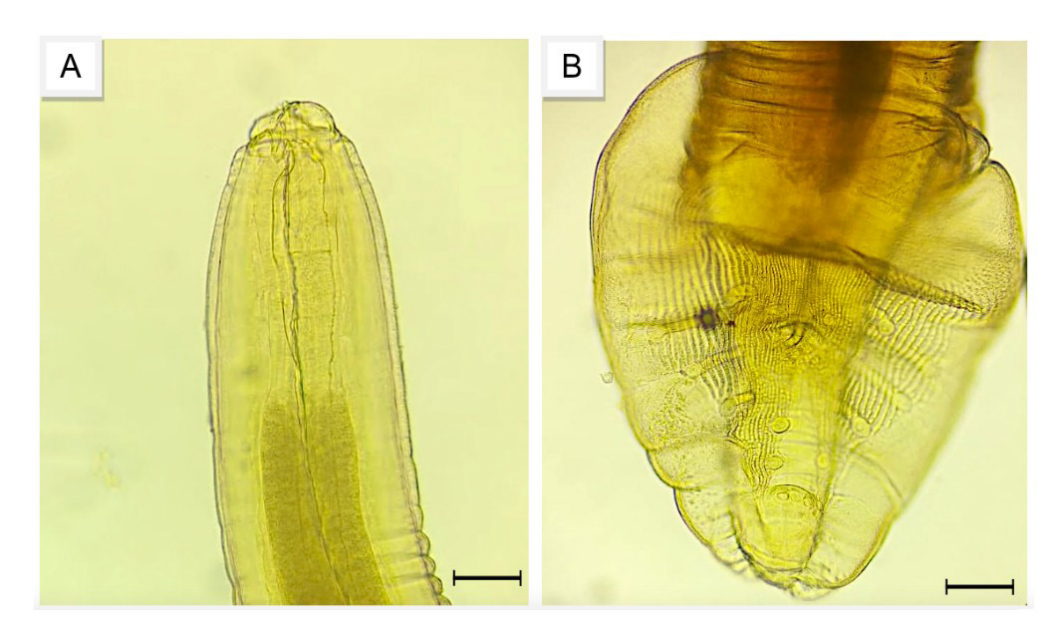
Adult specimens of nematodes found in fecal samples from maned wolves (*Chrysocyon brachyurus*) in Serra da Canastra National Park, MG. (A) Anterior end of an adult of *Physaloptera* sp., bar = 20 μm; (B) Posterior end of *Physaloptera* sp., bar = 20 μm.

Among the positive samples, 53.4% (16/30) corresponded to infections by a single parasite, while 46.7% (14/30) were infections by multiple parasites, specifically: 20% (6/30) by two parasite species, 20% (6/30) by three species, and 6.67% (2/30) by four species. Figures 2 and 3 illustrate parasite eggs and adults found in the fecal matter of maned wolves. The coproparasitological tests of fecal samples from maned wolves revealed a prevalence of 75% (30/40) for at least one parasite species, while domestic dogs showed a prevalence of 22.5% (9/40), with a statistically significant difference between the two host species (p=0.0001).

### Comparative diagnoses in dogs and maned wolves

A total of 80 fecal samples from canids were collected, with half from maned wolves inhabiting the PNSC and the other half from domestic dogs from the surrounding region of the Conservation Unit. Although the number of samples was equal for both host species, the results revealed significant differences after the coproparasitological analysis.

#### Parasitological results

The coproparasitological examinations of maned wolves showed a prevalence of 75% (30/40) of samples positive for at least one parasite species. In contrast, the prevalence of positive samples in dogs was 22.5% (9/40), with a statistically significant difference between them (p=0.0001). This indicates that the number of positive fecal samples from maned wolves was more than three times that of domestic dogs.

#### Frequency

Fecal matter from dogs showed a more uniform distribution of parasitism, with 25% (2/8) of samples testing positive during the dry season and 21.8% (7/32) in the rainy season. In contrast, fecal matter from maned wolves showed a parasite prevalence rate of 90.47% (19/21) in the dry season, and of 57.9% (11/19) in the rainy season. The statistical analysis revealed a significant difference in parasitism between maned wolves and dogs in the dry (p=0.0014) and rainy (p=0.0215) seasons.

#### Infection characteristics

In terms of parasitic infections, the dogs exhibited 88.9% (8/9) of single infections and 11.1% (1/9) of multiple infections, while the maned wolves presented 53.4% (16/30) of single infections and 46.6% (14/30) of multiple infections. In other words, the two host species showed no statistically significant difference (p=0.1152).

**Figure 4:**
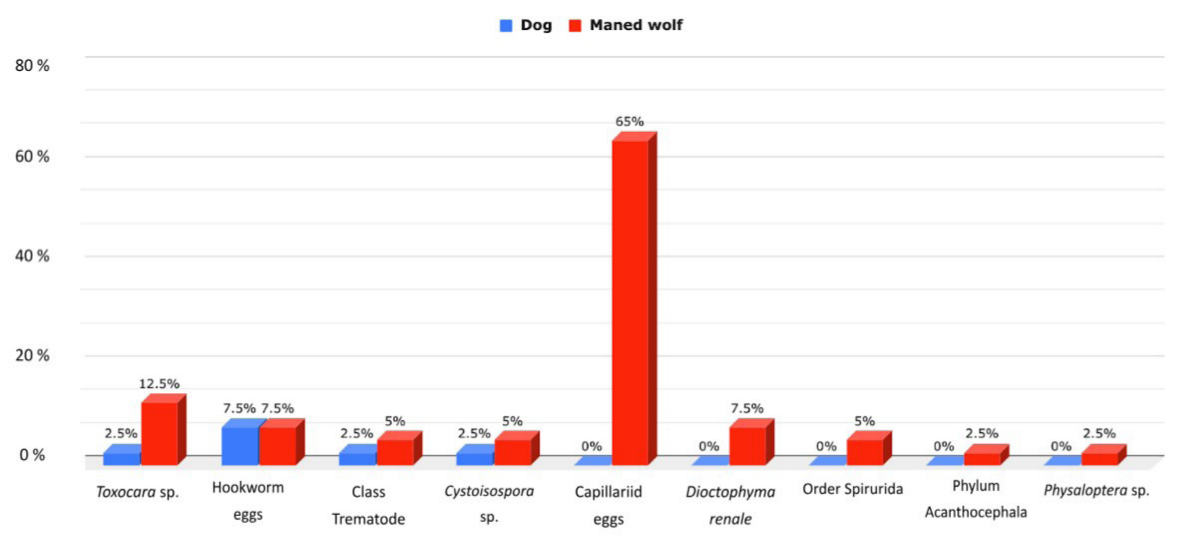
Comparative analysis of parasitic infections, by parasites, in maned wolves (*Chrysocyon brachyurus*) and domestic dogs in the region of Serra da Canastra National Park, MG.

All the parasite eggs and oocysts identified in the fecal samples from dogs were also found in those from maned wolves, but with different prevalence rates, although the fecal matter from maned wolves contained a greater variety of parasite species. A statistical analysis revealed no statistically significant difference between *Toxocara* sp. (p=0.9678) and *Cystoisospora* sp. (p=0.9908) infections in maned wolves and domestic dogs (Figure 4).

## Discussion

Non-invasive sample collection is particularly advantageous for species with nocturnal habits and low population density, such as carnivores, as it prevents stress and the negative impact associated with capture. The analysis of fecal material, for instance, can provide data on diet, hormones, ecological roles in seed dispersal and species control, as well as the dynamics of gastrointestinal parasites (Morin & Woodruff, 1996; Kohn & Wayne, 1997; Chame, 2003).

Other researchers have carried out coproparasitological tests on maned wolf and domestic dog feces with non-invasive sample collection in the Southeast and Central-West regions of Brazil, including Braga et al. (2010), Santos et al. (2012), Curi et al. (2012), Araújo (2014), Massara et al. (2015), Dib et al. (2020), and Marins et al. (2021).

Another relevant aspect is the time between defecation and sample collection. To minimize the degradation of eggs and larvae caused by climatic factors, the samples were collected a few hours after defecation, between 5:00 and 7:00 a.m., ensuring greater accuracy in parasite identification. This temporal control is essential to prevent false negatives resulting from the leaching of parasitic structures due to prolonged exposure to humidity, high temperatures, or solar radiation.

In a study conducted by Araújo (2014) in the private natural heritage reserve (PNHR) of Santuário do Caraça, MG, 28 fecal samples were collected from maned wolves and 30 from domestic dogs, with a prevalence rate of 42.85% (12/28) and 43.33% (13/30) respectively. The test results of dog feces collected in the PNHR of Santuário do Caraça are inconsistent with those of this study, which showed lower prevalence rates. In the case of dogs in the PNHR, the low rate of parasitism is probably due to the good general condition and adequate health management of these animals.

Although maned wolves and dogs share many sites in the PNHR area, they showed differences in parasitism rates. This may be attributed to specific characteristics of these canids, whose trophic levels, eating habits and range of geographic dispersion differ from one another.

The fecal samples from maned wolves collected in the PNHR revealed a prevalence rate of 75% (30/40) of positivity for at least one parasite species, most of them being parasitic helminths with some zoonotic potential. This suggests possible interactions between these animals and the existence of different degrees of anthropization in natural areas. The high prevalence of parasites in maned wolves may be associated not only with their population density in the Serra da Canastra National Park (PNSC), estimated at 0.072 individuals/km^2^ (Lemos et al., 2023), but also with their ecological role as top predators.

In another study conducted by Santos et al. (2012) in the region of Serra do Cipó National Park, MG, parasites of domestic dogs and maned wolves were compared, with fecal samples collected from 45 dogs, of which 28 (62.2%) tested positive for some type of parasite. The most frequent family was Ancylostomidae, found in 19 (42.2%) samples. Among the 33 fecal samples collected from maned wolves, 31 (93.9%) were positive for helminths, with the Trichuridae family being the most prevalent, occurring in 25 (75.8%) of the samples. Additionally, hookworm eggs, *Toxocara* sp., *Cystoisospora* sp., and acanthocephalan eggs were found in the maned wolf samples, as in our research.

It is important to emphasize that in Serra do Cipó National Park, domestic dogs and maned wolves live in close proximity due to the characteristics of the region, as these conservation units are surrounded by rural properties. Both canid species were also hosts to hookworm eggs, *Toxocara* sp., and *Cystoisospora* sp., as observed in our study (Santos et al., 2012).

The high prevalence of parasites in fecal samples from maned wolves found in this study is consistent with previous studies carried out in conservation units. An example of this is the survey conducted in Emas National Park, GO by Braga et al. (2010), who reported a prevalence rate of 87.7% (43/49) of fecal matter positive for parasites (*Ancylostoma caninum*, *Trichuris trichiura*, *Trichuris vulpis* and *Ascaris* sp.).

On the other hand, Curi et al. (2012) reported a prevalence of 100% (15/15) in the PNHR Serra do Galheiro, MG, which is the highest prevalence of positive fecal matter with hookworm eggs, trichurid eggs, acanthocephalans eggs and *Physaloptera* sp. data are also confirmed by the findings of Dib et al. (2020) in Itatiaia National Park, RJ, who described a prevalence rate of 81.4% (79/97) with capillariid eggs, trichurid eggs, ascarid eggs and *Physaloptera* sp.

Lower prevalence rates were also described by Araújo (2014) in the PNHR of Santuário do Caraça, MG, with 53.33% (15/28) of fecal matter positive for parasites (hookworm eggs, acanthocephalan eggs, *Capillaria* sp., *Trichuris* sp., *Toxocara* sp. and *Cystoisospora* sp.). Similarly, in a study also conducted in the PNSC, Marins et al. (2021) analyzed 103 fecal samples from maned wolves during the period of 2017 to 2019 and reported a prevalence of 45.63% (47/103) of positive samples. It should be noted that the parasites *Ancylostoma* sp., *Toxocara* sp., *Cystoisospora* sp. and trematode eggs were found in fecal samples from domestic dogs and from maned wolves in the PNSC.

In this study, the prevalence rate of positive samples in the dry season was almost twice that found during the rainy season. According to Paula & Gambarini (2013), the diet of the maned wolf varies throughout the year because it is an omnivorous canid. In the dry season, when fruit availability declines, the diet of the maned wolf consists predominantly of other animals, suggesting that most of the fecal samples from maned wolves were collected during a period when these animals were preying on other animals, such as rodents, birds and lizards.

The ingestion of infected prey can result in the presence of parasitic structures in feces without necessarily indicating an active infection, characterizing pseudoparasitism. This phenomenon occurs when parasite eggs or larvae are expelled in the feces of a predator after the ingestion of an intermediate or paratenic host, without the parasite's life cycle being completed in the definitive host.

Maned wolves living in the PNSC may be definitive hosts of parasites from intermediate hosts that serve as their prey or are temporary carriers of parasitic species from other hosts, without being infected (pseudoparasitism). A strong indication that supports this analysis is that an adult nematode was collected during the macroscopic examination of fecal matter from maned wolves. This parasite was identified as *Physaloptera* sp., a helminth found in the stomach of reptiles such as the tegu lizard (*Salvator merianae*).

The relevant discovery in this study was parasitism by *Dioctophyma renale* in maned wolves, which presented a prevalence of 10% (3/30). *D. renale* is a parasite of the urinary tract of canids, and the diagnosis is made by urine sedimentation test for the detection of eggs, and not by fecal tests. Generally, the definitive host is infected by ingesting oligochaete annelids (earthworms) or paratenic hosts (fish and frogs) or intermediate hosts. Infection by *D. renale* in Brazil was first described by Molin (1860), through necropsy of a maned wolf.

Parasitosis has been described in other wild canids around the world, such as coyotes (*Canis latrans*), foxes (*Canis vulpes*), wolves (*Canis lupus*), jackals (*Canis mesomelas*), and bush dogs (*Speothos venaticus*) (Barriga, 1982; Soulsby, 1982; Fortes, 2004; Foreyt, 2005; Leite et al., 2005). The presence of eggs of this nematode in maned wolf feces suggests the specific behavior of wolves to urinate on their own feces. The maned wolf uses its feces and urine to mark its territory, but there are no records in the literature of ethology studies of this species describing the act of urinating on feces.

It is noteworthy that most of the parasites identified in this research, such as capillariid eggs, hookworm eggs and *T. canis*, have a potential for zoonotic transmission. In fact, hookworm eggs and *T. canis* were present in the feces of both the host species under study. This suggests that these parasites may be circulating between the two host species, and are possibly being transmitted to humans, given the proximity of the PNSC to urbanized areas.

The higher prevalence of parasites diagnosed in *C. brachyurus* and *C. familiaris* is related to nematode infections of particular public health interest. This finding confirms the importance of these canids and their interrelationships with humans. Research on wildlife parasites, especially in carnivores and other secondary consumers, increases our understanding of the consequences for human health of interactions between wild and domestic animals, and our awareness of the real importance of each host species in the life cycle and transmission of parasites.

## Conclusions

The highest prevalence of parasites in the feces of maned wolves in Serra da Canastra National Park (PNSC) was capillariid eggs, with 86.6% (26/30) of samples testing positive, and the highest fecal parasite prevalence rates in maned wolves in PNSC were observed during the dry season. Additionally, the parasitic structures of hookworms, *Toxocara* sp. and *Cystoisospora* sp. showed a morphological similarity in the two canid species of this study. The fecal tests performed here suggest that the two host species share and exchange parasites. We can suggest that the PNSC harbors essential elements for the maintenance of complex parasitic life cycles in maned wolves, which include various hosts, such as intermediate and paratenic hosts, facilitating parasitic infections in animals or cases of pseudoparasitism. Therefore, parasitic epidemiological surveillance is necessary in this region.
